# A blood-based biomarker panel indicates IL-10 and IL-12/23p40 are jointly associated as predictors of β-amyloid load in an AD cohort

**DOI:** 10.1038/s41598-017-14020-9

**Published:** 2017-10-25

**Authors:** Steve Pedrini, Veer B. Gupta, Eugene Hone, James Doecke, Sid O’Bryant, Ian James, Ashley I. Bush, Christopher C. Rowe, Victor L. Villemagne, David Ames, Colin L. Masters, Ralph N. Martins, AIBL Research Group, Greg Savage, Bill Wilson, Pierrick Bourgeat, Jurgen Fripp, Simon Gibson, Hugo Leroux, Simon McBride, Olivier Salvado, Michael Fenech, Maxime Francois, Mary Barnes, Jenalle Baker, Kevin Barnham, Shayne Bellingham, Julia Bomke, Sveltana Bozin Pejoska, Rachel Buckley, Lesley Cheng, Steven Collins, Ian Cooke, Elizabeth Cyarto, David Darby, Vincent Dore, Denise El-Sheikh, Noel Faux, Christopher Fowler, Karra Harrington, Andy Hill, Malcolm Horne, Gareth Jones, Adrian Kamer, Neil Killeen, Hannah Korrel, Fiona Lamb, Nicola Lautenschlager, Kate Lennon, Qiao-Xin Li, Yen Ying Lim, Andrea Louey, Lance Macaulay, Lucy Mackintosh, Paul Maruff, Alissandra Mcilroy, Julie Nigro, Kayla Perez, Kelly Pertile, Carolina Restrepo, Barbara Rita Cardoso, Alan Rembach, Blaine Roberts, Jo Robertson, Rebecca Rumble, Tim Ryan, Jack Sach, Brendan Silbert, Christine Thai, Brett Trounson, Irene Volitakis, Michael Vovos, Larry Ward, Andrew Watt, Rob Williams, Michael Woodward, Paul Yates, Fernanda Yevenes Ugarte, Ping Zhang, Sabine Bird, Belinda Brown, Samantha Burnham, Pratishtha Chatterjee, Kay Cox, Shane Fernandez, Binosha Fernando, Sam Gardener, Simon Laws, Florence Lim, Lucy Lim, Michelle Tegg, Kathy Lucas, Georgia Martins, Tenielle Porter, Stephanie Rainey-Smith, Mark Rodrigues, KaiKai Shen, Harmid Sohrabi, Kevin Taddei, Tania Taddei, Sherilyn Tan, Giuseppe Verdile, Mike Weinborn, Maree Farrow, Shaun Frost, David Hanson, Maryam Hor, Yogi Kanagasingam, Wayne Leifert, Linda Lockett, Malcolm Riley, Ian Saunders, Philip Thomas

**Affiliations:** 1 School of Medical Sciences, Edith Cowan University, Joondalup, WA 6027 Australia; 2 Co-operative Research Centre for Mental Health, Carlton, VIC 3053 Australia; 6 The Florey Institute, The University of Melbourne, Parkville, VIC 3052 Australia; 7 Department of Nuclear Medicine and Centre for PET, Austin Health, Heidelberg, VIC 3084 Australia; 8 National Ageing Research Institute, Parkville, VIC 3052 Australia; 12 Department of Psychology, Macquarie University, Sydney, NSW 2109 Australia; 13 CSIRO, North Ryde, NSW 2113 Australia; 14 CSIRO, Herston, QLD 4029 Australia; 15 CSIRO, Adelaide, SA 5000 Australia; 16 Bio21 Institute of Molecular Science and Biotechnology, The University of Melbourne, Parkville, VIC 3052 Australia; 17 The University of Melbourne, Parkville, VIC 3052 Australia; 18 CSIRO, Melbourne, VIC Australia; 19 Cogstate Ltd., Melbourne, VIC 3000 Australia; 20 Alzheimer Australia Vic, Hawthorne, VIC 3122 Australia; 21 St. Vincent Hospital, Fitzroy, VIC 3065 Australia; 22 CSIRO, Floreat, WA 6014 Australia; 23 University of Western Australia, Crawley, WA 6009 Australia; 24 School of Biomedical Sciences, Curtin University, Bentley, WA 6102 Australia; 3 CSIRO Digital Productivity Flagship, Brisbane, QLD 4029 Australia; 4 University of North Texas Health Science Center, Fort Worth, 76107 Texas USA; 5 Institute for Immunology & Infectious Diseases, Murdoch University, Murdoch, WA 6150 Australia; 9 Academic Unit for Psychiatry of Old age, St. George’s Hospital, The University of Melbourne, Parkville, VIC 3052 Australia; 10 School of Psychiatry and Clinical Neurosciences, University of Western Australia, Crawley, WA 6009 Australia; 11 Department of Biomedical Sciences, Faculty of Medicine and Health Sciences, Macquarie University, Sydney, NSW 2109 Australia

## Abstract

Alzheimer’s Disease (AD) is the most common form of dementia, characterised by extracellular amyloid deposition as plaques and intracellular neurofibrillary tangles of tau protein. As no current clinical test can diagnose individuals at risk of developing AD, the aim of this project is to evaluate a blood-based biomarker panel to identify individuals who carry this risk. We analysed the levels of 22 biomarkers in clinically classified healthy controls (HC), mild cognitive impairment (MCI) and Alzheimer’s participants from the well characterised Australian Imaging, Biomarker and Lifestyle (AIBL) study of aging. High levels of IL-10 and IL-12/23p40 were significantly associated with amyloid deposition in HC, suggesting that these two biomarkers might be used to detect at risk individuals. Additionally, other biomarkers (Eotaxin-3, Leptin, PYY) exhibited altered levels in AD participants possessing the APOE ε4 allele. This suggests that the physiology of some potential biomarkers may be altered in AD due to the APOE ε4 allele, a major risk factor for AD. Taken together, these data highlight several potential biomarkers that can be used in a blood-based panel to allow earlier identification of individuals at risk of developing AD and/or early stage AD for which current therapies may be more beneficial.

## Introduction

Alzheimer’s Disease (AD) is the most common form of dementia in the elderly, characterised by the accumulation of extracellular senile plaques and intracellular neurofibrillary tangles in the brain^1^. Senile plaques are mainly composed of amyloid-β (Aβ), which is a product of Amyloid Precursor Protein (APP) that can undergo amyloidogenic cleavage to produce Aβ, or non-amyloidogenic cleavage to produce a p3 fragment^2,3^. Brain functions decline with disease progression as consequence of synaptic loss and neuronal death, ultimately reducing the brain volume^4–7^. Currently, there is no cure for AD and current treatments are aimed at reducing the symptoms and rate of decline, rather than addressing underlying causes of the disease^8,9^. It has been proposed that earlier interventions for AD may be more effective but it is obvious that the diagnostic process must be improved to detect the disease either before its onset or in its early stages. As of today, definitive diagnosis of the disease can only be achieved post-mortem by examination of the brain tissue. While other analysis are available, they are not suitable for large scale screening. For instance, it is possible to detect amyloid deposition through the use of PET scans using Pittsburgh Compound B (PiB) as the radiotracer, which has proven to be effective in identifying individuals who are at risk before AD onset since the technique has demonstrated that Aβ deposition takes place years before the clinical onset^10^. However, this analysis is quite expensive, requires specially trained physicians and bulky machinery, which means that it cannot be used to perform large scale testing. An alternative test is the analysis of the cerebrospinal fluid (CSF). While this analysis has provided positive results in discriminating between healthy controls (HC) and AD with several biomarker panels having been proposed^11–15^, it also requires trained operators and inherently carries more risk. The collection of CSF cannot be performed too often and cannot be routinely performed to screen a large population. Because of the risks and invasiveness, many individuals would refuse to undergo such a procedure, limiting the pool of available samples. Ideally, a blood-based test for AD using serum or plasma would be the best choice, as it would be inexpensive, relatively non-invasive, widely accessible with low associated risk and sampling could be performed almost anywhere. However, despite current efforts, there is no blood test that can diagnose AD. In the recent years several groups have attempted to create a biomarker panel that would predict the transition to disease in healthy individuals or to differentiate between AD and other forms of dementia. In 2007 Ray and collaborators^16^ suggested a blood-based biomarker panel of 18 proteins that was able to predict the conversion to AD 2–6 years later. As this panel was considered a milestone in the field, other groups have subsequently attempted to confirm these results using different cohorts. In 2008, another report indicated that a panel of 5 proteins from the former 18-protein panel was sufficient to distinguish controls from AD with the same accuracy^17^. In subsequent studies of the former 18-protein panel, only 3 and 5 proteins were found to be associated with AD, respectively^18,19^. Our group has recently published an 18-protein panel which was able to distinguish between healthy controls and AD^20^, while others have performed similar analysis in their cohorts^21–24^. Some groups have suggested panels of 3, 4 or 11 proteins that were able to distinguish between AD and controls^25–27^. A common denominator in all these studies is the heterogenetic time-points samples were collected with regards to the disease, the biomarkers evaluated and the statistical analysis used to validate effective panels. In our analysis we evaluated 22 biomarkers at 2 time points, in order to determine their association with the disease both cross-sectionally and longitudinally. This allowed us to examine whether any of these biomarkers were predictive of the conversion from healthy controls to MCI or AD and to determine their association with Aβ deposition in the cognitively healthy brain. Together, this analysis provides additional data which could help in finding a specific biomarker panel to distinguish between healthy and AD participants and/or identify those at risk of developing AD.

## Materials and Methods

### The AIBL Cohort

The AIBL study was approved by the ethics committees of St. Vincent’s Health, Hollywood Private Hospital, Austin Health and Edith Cowan University (Australia). All methods were performed in accordance with the relevant guidelines and regulations. A total of 1176 individuals were enrolled in the AIBL study and all volunteers gave written and informed consent before participating in our study. AIBL is a prospective longitudinal study in which healthy controls and patients are evaluated every 18 months. Individuals were evaluated in the morning, after an overnight fast. During each assessment, several body parameters, including weight, blood pressure and pulse rate were recorded, after which blood was drawn and collected in EDTA tubes for subsequent processing and analysis of the plasma^28^. Cognitive evaluations were then performed^29,30^. A more detailed description of the recruitment process has been previously described^28^. The diagnostic classifications were performed in accordance with the NINCDS-ARDA criteria^31,32^. For this study a total of 665 participants whom had their blood drawn at 18 and 54 months were included.

### Blood collection and *APOE* genotype

Plasma was isolated from whole blood collected in EDTA tubes by centrifugation, aliquot and stored at −80 °C. *APOE* status was determined by genotyping cells from whole blood as previously described^33^.

### Plasma biomarker Assay

22 analytes (IL-1α, IL-1β, IL-5, IL-6, IL-7, IL-10, IL-12/23p40, IL-13, IL-15, IL-17, EGF, EGFR, Eotaxin-3, Leptin, Angiopoietin-2, MCP-1, MMP-2, MIP-1α, PYY, SCF, TARC and TNF-α) were measured using custom assays (MesoScale Diagnostics, Maryland, USA). Briefly, these analytes were spread across three multiplex assay panels (5-plex: Angiopoietin-2, SCF, EGFR, Leptin and PYY, sample dilution: 2-fold; 8-plex: EGF, IL-1α, IL-12/23p40, IL-13, IL-15, IL-17, MCP-1 and MMP-2, sample dilution: 2-fold; 9-plex: Eotaxin-3, IL-1β, IL-5, IL-6, IL-7, IL-10, MIP-1α, TARC and TNF-α, sample dilution: undiluted) where analytes were grouped together based on their dilution, antibody compatibility and optimal assay condition requirements as specified by the manufacturer. Blocking the plate (if required) was performed with buffer provided by the manufacturer, samples were diluted according to manufacturer’s instructions and incubated at room temperature for 2 hours or overnight at 4 °C, depending on the assay panel. After washing the plates 3 times with PBST (pH 7.4), the specified detection antibodies were added and plates were incubated for a further 1 hour at room temperature, followed by 3 washes with PBST. Read buffer was then added and plates were read on the SECTOR Imager (MSD, Maryland, USA).

### PET scan

A selected number of individuals of the AIBL cohort (at 18 months HC PiB−, n = 94 and HC PiB+, n = 27; at 54 months HC PiB−, n = 67 and HC PiB+, n = 26) volunteered for Positron Emission Tomography with the labelled Pittsburgh compound B (^11^C-PiB−PET) to measure cerebral amyloid load^34^. PiB score, expressed as Standardized Uptake Value Ratio (SUVR), was calculated by normalizing the Standardized Uptake Value (SUV) to the cerebellar SUV. The common SUVR threshold of 1.5 was used to demarcate individuals with low amyloid deposition (PiB−, SUVR < 1.5) or high amyloid deposition (PiB+, SUVR > 1.5)^10^.

### Statistical analysis

Statistical comparisons of markers at either 18 months or 54 months were carried out using linear models correcting for age, site, *APOE* ε4 allele status and gender. Results were considered significant when p < 0.05. The combined analysis over both time points simultaneously was performed using mixed modelling to accommodate correlations between observations on the same individual. Logistic regression analysis was used to determine the joint association between multiple biomarkers and group outcomes. All calculations were carried out using TIBCO Spotfire S^+^ ver 8.2 statistical software (TIBCO Software, Inc., Boston, MA). Note that while data in the tables are presented as pg/ml, statistical analysis was performed using the log_10_ transformation of the raw data to better approximate normality. For IL-5, IL-6 and IL-17 the log_10_ transformation was performed on the (raw data + 1) in order to accommodate the 0 values in the dataset. ROC analysis for prediction of high or low SUVR values was carried out via logistic regression comparing a base model which included age + sex + *APOE* genotype and an extended model including age + sex + *APOE* genotype + log(IL-10*IL-12/23).

## Results

### Demographics

The basic demographic of this study are summarized in Table 1. A total of 559 healthy controls (HC), 39 mild cognitive impaired (MCI) and 67 Alzheimer patients (AD) were evaluated. Plasma samples were analysed for all biomarkers listed. However, in spite of analysing our biomarker panel in all individuals, the difference in available data for each biomarker are due to the difference in CV across duplicates, which lead us to eliminate duplicate values with high CV. The elimination rate was different for each biomarker analysed. We used a 30% CV cut-off above which the calculated value was considered not suitable for analysis. While this value may appear high, it should be noted that cytokines levels were extremely low and often fell in the lowest section of the standard curve, where small differences in the raw value resulted in a much larger difference in the calculated result. Hence, while a low percent CV of the raw value, which would normally indicate good duplicates in the assay may translate into high CV of the calculated result. In order to keep the analysis consistent, when a sample at either 18 or 54 months was not available, its counterpart at the other time point was also removed, even if the sample fell in the readable range. Of the 22 biomarkers evaluated, IL-13 was excluded as duplicates displayed inconsistent reproducibility while IL-1α, IL-1β and MIP-1α levels were below the detectible range for most of the samples. In Table 2 we report the valid cases for each individual biomarker at both time points. In order to demonstrate that the removal of samples with high CV did not affect the average biomarker values, all samples regardless of their CV were tabulated (Supplementary Table 1). As shown, the overall averages of all samples (Supplementary Table 1) are similar to the average values of the valid cases (Table 3). Therefore the exclusion of these samples did not affect the average values of these biomarkers.

**Table 1:** Demographic data for our cohort. (a) Data are represented as Mean ± S.E or as number of cases overall.

	HC	MCI	AD
Age at baseline	69 ± 6	75 ± 6	76 ± 7
Gender (M/F) at 18 months	234/325	22/17	23/44
Site (Mel/Per) at 18 months	327/232	22/17	47/20
*APOE* ε4 carrier (y/n) at 18 m	146/413	17/22	47/20
n at 18 months /54 months	559/528	39/51	67/86

**Table 2:** Individual number of cases analysed for each single biomarker in our 3 groups (HC, MCI, AD).

	HC 18	MCI 18	AD 18	Total 18	HC 54	MCI 54	AD 54	Total 54
EGF	541	38	65	644	512	49	83	644
IL12/23 p40	492	35	60	587	468	42	77	587
IL-15	554	38	66	658	523	51	84	658
IL-17	396	29	47	472	374	37	61	472
MCP-1	529	38	60	627	501	47	79	627
MMP-2	549	38	64	651	519	49	83	651
Eot-3	510	35	61	606	482	45	79	606
IL-5	449	31	57	537	421	45	71	537
IL-6	472	35	58	565	444	45	76	565
IL-7	552	38	66	656	521	51	84	656
IL-10	550	39	67	656	520	50	86	656
TARC	507	34	57	598	479	45	74	598
TNF-a	552	39	56	657	521	51	85	657
Ang-2	535	38	56	639	505	49	85	639
SCF	501	37	64	602	472	47	83	602
EGFR	534	38	66	638	504	49	85	638
Leptin	528	38	66	632	499	48	85	632
PYY	363	28	49	440	338	41	61	440

### Longitudinal assessment of biomarkers

Table 3 illustrates changes in biomarkers at 2 different time points, 18 months and 54 months in non-converters, with p-values corrected for age, gender, site and *APOE* ε4 carriage. PYY levels were significantly higher in AD at 18 months (p = 0.02) but not at 54 months (p = 0.09). In contrast Eotaxin-3 levels were significantly higher in AD at 54 months (p = 0.03) but not at 18 months (p = 0.39). EGF and TARC levels showed increased levels in AD at 54 months (p = 0.08 for both) but not at 18 months (p = 0.48 and p = 0.62, respectively). A mixed model analysis combining the two time points (Table 4) found that only Eotaxin-3, TARC and PYY levels showed a trend (p = 0.07, p = 0.06 and p = 0.07, respectively). The p-values reported for these were for single analyte analysis, and are not significant upon Bonferroni’s correction for multiple comparisons (data not shown).

**Table 3:** General Linear Model analysis of the biomarkers in HC and AD at 18 and 54 month time points in non-converters only. Data are presented as Mean ± S.E., although statistical analysis was performed on the log_10_ value to better approximate a normal distribution. For IL-5, IL-6 and IL-17 the log_10_ conversion was performed on the (raw data + 1) in order to accommodate 0 values.

	HC 18	AD 18	p	HC 54	AD 54	p
EGF	27.33 ± 1.69	28.81 ± 6.52	0.48	16.41 ± 0.57	22.46 ± 2.48	*0.08*
IL12/23 p40	114.47 ± 2.88	119.81 ± 9.39	0.20	122.01 ± 3.59	143.64 ± 11.45	0.60
IL-15	1.96 ± 0.02	2.14 ± 0.07	0.11	2.26 ± 0.03	2.52 ± 0.08	0.21
IL-17	1.59 ± 0.10	1.82 ± 0.22	0.29	2.15 ± 0.46	2.20 ± 0.27	0.37
MCP-1	62.46 ± 0.88	70.99 ± 5.85	0.44	67.96 ± 1.06	76.37 ± 3.79	0.44
MMP-2	83561 ± 794	86564 ± 2690	0.78	89516 ± 894	94120 ± 3072	0.67
Eot-3	4.80 ± 0.67	8.47 ± 3.20	0.39	6.81 ± 2.84	7.72 ± 2.51	**0.03**
IL-5	0.74 ± 0.08	0.48 ± 0.05	0.26	0.67 ± 0.05	0.54 ± 0.06	0.61
IL-6	1.38 ± 0.30	1.39 ± 0.20	0.32	1.84 ± 0.42	1.59 ± 0.19	0.21
IL-7	0.99 ± 0.06	0.90 ± 0.11	0.76	0.64 ± 0.03	0.77 ± 0.06	0.16
IL-10	1.30 ± 0.14	1.05 ± 0.05	0.49	1.26 ± 0.11	1.44 ± 0.34	0.78
TARC	72.15 ± 5.26	77.68 ± 11.09	0.62	54.92 ± 2.94	68.64 ± 7.21	*0.08*
TNF-a	1.62 ± 0.03	1.86 ± 0.09	0.54	1.70 ± 0.03	2.11 ± 0.12	0.13
Ang-2	8031 ± 188	8553 ± 493	0.83	8897 ± 210	9592 ± 594	0.60
SCF	92.70 ± 1.24	92.63 ± 3.60	0.50	97.82 ± 1.42	101.21 ± 4.23	0.96
EGFR	34396 ± 311	33559 ± 785	0.43	34625 ± 301	32745 ± 809	0.71
Leptin	27930 ± 1787	29764 ± 4993	0.59	31358 ± 2131	43674 ± 7188	0.42
PYY	79.65 ± 1.98	103.06 ± 8.97	**0.02**	85.94 ± 2.34	106.69 ± 9.91	*0.09*

**Table 4:** Combined analysis of the biomarkers with longitudinal assessment between HC and AD.

	Combined analysis p value
EGF	0.39
IL12/23p40	0.54
IL-15	0.47
IL-17	0.28
MCP-1	0.81
MMP-2	0.25
Eot-3	*0.07*
IL-5	0.33
IL-6	0.22
IL-7	0.23
IL-10	0.74
TARC	*0.06*
TNF-a	0.35
Ang-2	0.19
SCF	0.23
EGFR	0.69
Leptin	0.71
PYY	*0.07*

### Eotaxin-3, Leptin and PYY show altered levels in a subset of AD patients (*APOE* ε4 carrier)

In Tables 5 and 6 we evaluated 5 biomarkers (IL-12/23p40, Eotaxin-3, Angiopoietin-2, Leptin and PYY) that had shown differences based on their *APOE* genotype by subdividing the non-converters group into *APOE* ε4− and *APOE* ε4+. In the *APOE* ε4− group (Table 5) IL-12/23p40 is slightly lower in AD at 18 months only (p = 0.08), while Leptin levels tend to be lower, although not significantly, in AD versus HC at both time points (p = 0.13 and p = 0.17, respectively). However, when evaluating the same biomarkers in the *APOE* ε4+ group (Table 6), Eotaxin-3, Leptin and PYY levels are higher in AD patients at both assessments. Although the mixed model analysis did not show any statistical significance overall (Table 7), both Leptin and PYY levels are significantly higher in AD/*APOE* ε4+ at 54 months compared to the other groups (Leptin p = 0.035, PYY p = 0.0013). We also performed the General Linear Model analysis in the AD group only comparing Leptin and PYY in *APOE* ε4− vs *APOE* ε4+ at both time points. The analysis did not show significance for Leptin (p = 0.18 and p = 0.13 at 18 and 54 months, respectively), neither for PYY (p = 0.77 and p = 0.33 at 18 and 54 months, respectively). However, the analysis itself may have been affected by the low number of cases (PYY: n = 16 and 33 for *APOE* ε4− and *APOE* ε4+, respectively and Leptin: n = 19 and 47 for *APOE* ε4− and *APOE* ε4+, respectively). Although it would have been interesting to examine the effect of participants being heterozygous or homozygous for the *APOE* ε4 allele, due to the very small sample size of *APOE* ε4 homozygous participants (for PYY n = 26 of *APOE* ε4 heterozygous and n = 7 of *APOE* ε4 homozygous; for Leptin n = 36 of *APOE* ε4 heterozygous and n = 11 of *APOE* ε4 homozygous) in the AD group, undertaking such an analysis was not feasible.

**Table 5:** General Linear Model analysis of 5 biomarkers in HC and AD at 18 and 54 month time points in *APOE* ε4− in non-converters only. Data are presented as Mean ± S.E., although statistical analysis was performed on the log_10_ value to better approximate a normal distribution.

	HC 18	AD 18	p	HC 54	AD 54	p
IL12/23 p40	115.26 ± 3.44	105.81 ± 15.31	0.08	123.30 ± 4.40	126.74 ± 15.32	0.85
Eot-3	5.31 ± 0.89	3.16 ± 0.22	0.84	8.00 ± 3.79	4.03 ± 0.56	0.36
Ang-2	8135 ± 214	8481 ± 1082	0.69	8998 ± 242	8729 ± 1304	0.22
Leptin	28524 ± 2082	15472 ± 4401	0.13	33456 ± 2652	16863 ± 4490	0.17
PYY	80.36 ± 2.30	86.36 ± 7.60	0.45	86.26 ± 2.58	79.68 ± 6.15	0.53

**Table 6:** General Linear Model analysis of 5 biomarkers in HC and AD at 18 and 54 month time points in *APOE* ε4+ in non-converters only. Data are presented as Mean ± S.E., although statistical analysis was performed on the log_10_ value to better approximate a normal distribution.

	HC 18	AD 18	p	HC 54	AD 54	p
IL12/23 p40	112.08 ± 5.08	126.29 ± 11.76	0.84	118.09 ± 5.56	151.47 ± 15.13	0.36
Eot-3	3.28 ± 0.15	10.88 ± 4.61	0.41	3.26 ± 0.13	9.39 ± 3.62	0.06
Ang-2	7710 ± 387	8582 ± 544	0.71	8587 ± 428	9941 ± 649	0.80
Leptin	26110 ± 3474	35542 ± 6624	0.66	24928 ± 2935	54513 ± 9511	0.10
PYY	77.13 ± 3.83	111.15 ± 12.65	0.15	84.82 ± 5.44	119.78 ± 13.91	0.06

**Table 7:** Combined analysis of the 5 biomarkers with longitudinal assessment between HC and AD according to the *APOE* genotype.

	Combined analysis *APOE* ε4− p value	Combined analysis *APOE* ε4+ p value	AD/*APOE* ε4+ at 54 months vs the field p value
IL12/23 p40	0.28	0.99	
Eot-3	0.65	0.12	
Ang-2	0.24	0.55	
Leptin	0.09	0.60	**0.035**
PYY	0.93	0.17	**0.0013**

### IL-10 and IL-12/23p40 are jointly associated as predictors of β–amyloid load

**Figure 1:**
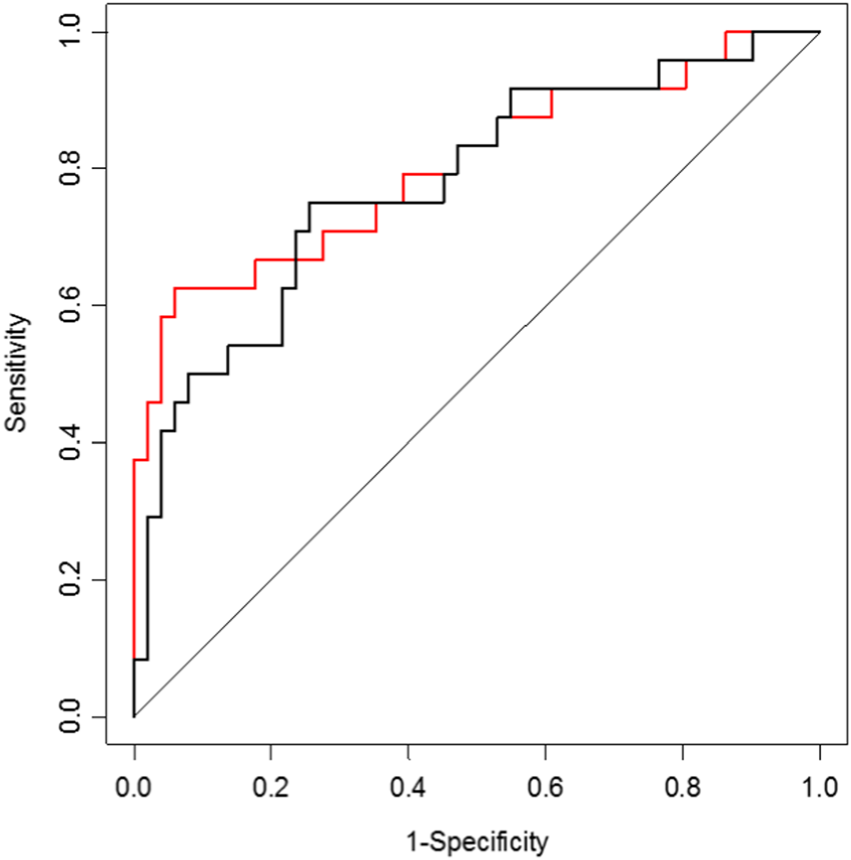
ROC analysis performed at 54 months. The black line represent the base model (age + sex + *APOE* ε4), while the red line represents the base model + log(IL-10*IL-12/23p40).

In order to determine whether these biomarkers can be useful diagnostically or predictively for development of AD, we analysed the biomarker levels in all HC (non-converters and converters) who had undergone amyloid imagining and stratified them into PiB− (SUVR < 1.5) or PiB+ (SUVR > 1.5). The results in Table 8 illustrate that only IL-12/23 p40 levels at 54 months were significantly higher in HC PiB+ versus HC PiB− (p = 0.023), while their increase at 18 months was not (p = 0.11). Similarly, IL-10 also showed a trend for increased levels in HC PiB+ at both time points (p = 0.13 and p = 0.15). No altered levels of any other biomarkers were associated with HC in which amyloid deposition is present (PiB+). The p-values reported here are performed for single analyte analysis and they are not significant upon Bonferroni’s correction for multiple comparisons (data not shown). Logistic regression analysis of the two markers jointly with PiB+ level as the outcome indicated that they were jointly significant with the effect restricted to the (log) product of the two markers (Table 9, p = 0.039 at 18 months and p = 0.017 at 54 months). ROC analysis performed at 54 months indicated an increased area under curve (AUC = 0.805) when including the IL-10*IL-12/23p40 to the base model (age + sex + APOE ε4) for which the AUC = 0.779 (Fig. 1). Similar results were obtained when evaluating our HC at 18 months (Base model AUC = 0.787; Base model + log(IL-10*IL-12/23p40) AUC = 0.802, data not shown).

**Table 8:** General Linear Model analysis of the biomarkers in HC according to brain amyloid deposition (PiB−, SUVR < 1.5 and PiB+, SUVR > 1.5) at 18 and 54 month time points. Data are presented as Mean ± S.E., although statistical analysis was performed on the log_10_ value to better approximate a normal distribution. For IL-5, IL-6 and IL-17 the log_10_ conversion was performed on the (raw data + 1) in order to accommodate 0 values.

	HC PiB− 18	HC PiB+ 18	p	HC PiB− 54	HC PiB+ 54	p
EGF	30.46 ± 3.38	25.37 ± 3.57	0.54	19.78 ± 2.00	18.10 ± 1.89	0.93
IL12/23 p40	104.81 ± 6.54	130.15 ± 24.06	*0.11*	105.67 ± 7.35	151.73 ± 24.61	**0.023**
IL-15	1.91 ± 0.06	1.97 ± 0.11	0.98	2.29 ± 0.08	2.34 ± 0.17	0.46
IL-17	1.44 ± 0.17	1.55 ± 0.32	0.91	1.80 ± 0.27	2.38 ± 1.02	0.90
MCP-1	66.24 ± 2.75	71.37 ± 4.47	0.16	70.27 ± 2.30	76.42 ± 4.03	0.33
MMP-2	81457 ± 1661	82594 ± 3356	0.54	87571 ± 2339	91574 ± 3226	0.57
Eot-3	5.08 ± 1.48	3.58 ± 0.47	0.46	5.36 ± 1.89	3.79 ± 0.44	0.93
IL-5	0.90 ± 0.22	0.65 ± 0.12	0.53	0.67 ± 0.12	0.67 ± 0.11	0.85
IL-6	1.11 ± 0.13	3.10 ± 1.90	0.48	1.06 ± 0.12	4.22 ± 2.45	0.29
IL-7	1.17 ± 0.12	0.94 ± 0.12	0.45	0.85 ± 0.12	0.74 ± 0.07	0.77
IL-10	1.20 ± 0.19	3.70 ± 2.40	*0.13*	1.01 ± 0.07	2.98 ± 1.78	*0.15*
TARC	69.29 ± 5.99	69.90 ± 6.93	0.72	57.89 ± 5.46	58.62 ± 6.98	0.72
TNF-a	1.60 ± 0.07	1.73 ± 0.13	0.81	1.59 ± 0.06	1.83 ± 0.13	0.57
Ang-2	7966 ± 461	8741 ± 660	0.64	7944 ± 374	8711 ± 614	0.74
SCF	94.56 ± 3.10	107.14 ± 5.04	0.26	101.14 ± 3.66	110.52 ± 5.53	0.99
EGFR	35064 ± 723	31726 ± 1285	0.18	36222 ± 773	33255 ± 1467	0.32
Leptin	34551 ± 6605	17707 ± 4844	0.24	34021 ± 6551	28271 ± 8892	0.35
PYY	76.69 ± 4.80	69.00 ± 5.98	0.55	85.58 ± 4.60	76.74 ± 8.81	0.54

**Table 9:** Logistic regression analysis for IL-10 and IL-12/23p40 in PiB+ and PiB− HC.

	18 months	54 months
IL-10*IL-12/23p40 (logistic regression)	**0.039**	**0.017**

We also evaluated the levels of the same biomarkers in all HC at 18 months based on their classification at 54 months, comparing the non-converter participants, who did not develop AD at 54 months, versus those who did convert to MCI or AD (data not shown). Here, at 18 months IL-12/23p40 and IL-10 levels tended to be higher in the converter group versus the non-converters. However, the low sample size of converters may be a constraint to the statistical analysis.

## Discussion

AD is the leading cause of dementia in the elderly, but current methods of ante-mortem diagnosis lack accuracy and specificity. Developing a biomarker panel that can provide early diagnosis of AD or indicate level of risk in healthy individuals remains a considerable challenge. Many studies have reported biomarker panels of 10 or more biomarkers but tended to be cross-sectional. Our study is longitudinal with 22 biomarkers. It was performed on the well characterised AIBL cohort, using biomarkers that have previously been associated with AD in previous studies. In order to provide a more detailed evaluation, we have assessed biomarkers at 2 different time points to determine whether biomarker changes were consistent over time, or specific to the early or late stages of the disease.

We evaluated biomarkers in non-converters, individuals whose cognition was unchanged at both time points. As shown in Table 3, only 4 biomarkers displayed altered levels between HC and AD. We found that PYY was significantly higher in AD individuals at 18 months and follows a similar trend at 54 months. PYY is a molecule that belongs to the pancreatic polypeptide family and has been linked to aluminium metabolism in AD^35^. Previous work did not show any differences in blood levels of PYY between controls and AD^36^.

Similarly, Eotaxin-3 significantly increased in AD at 54 months but not at 18 months. Other studies have reported that higher levels of Eotaxin-3 in plasma and CSF were observed and were associated with prodromal AD^11,24,37^. In our biomarker panel, 2 more analytes (EGF and TARC) appeared to be associated with AD. For instance, a decrease in EGF levels has previously been associated with AD in a study with a biomarker panel of 18 proteins to predict the conversion from MCI to AD^16^. However, subsequent studies using the same 18-protein panel failed to validate it as only 3 and 5 of those proteins were significantly associated with AD^18,19^. While EGF was significantly associated with AD in both studies, its levels in the AD participants were increased rather than decreased. These data corroborates with another study that also showed increased EGF levels in AD^27^. Our data indicated a trend towards increased EGF at 54 months but not at 18 months. However, this difference was mostly due to a more severe drop of EGF levels in the HC, rather than an increase in the AD group (Table 3).

TARC, also known as CCL17, is a chemokine constitutively expressed in thymus whose natural ligand is CCR4^38^. Similar to EGF, our data indicated a significant increase of TARC at 54 month but not at 18 months. While it can be argued that these biomarkers are likely more related to later stage AD, we have also observed that age and site were affecting biomarker levels. However, all our analysis were corrected for age, gender, *APOE* genotype and site. The combined mixed model analysis (Table 4), which also accounted for longitudinal changes showed that Eotaxin-3, TARC and PYY may represent a suitable stable biomarker for differentiating between HC and AD (p = 0.07, p = 0.06 and p = 0.07, respectively). While the overall analysis provides a broad picture that can be used to find a biomarker panel with stable analytes over time for the detection of AD, it is always important to remember that *APOE* genotype is currently a major risk factor for AD, therefore the presence of the ε4 allele must always be considered when evaluating biomarkers.

We have therefore evaluated 5 biomarkers that demonstrated clear differences according to *APOE* ε4 status. The data show consistent increases in plasma Eotaxin-3 levels in AD individuals carrying *APOE* ε4 at both time points. However, a large variability was also observed amongst these individuals, suggesting that the presence of the ε4 allele could increase Eotaxin-3, but was not sufficient by itself to cause this increase. Other factors that may be involved in some but not all of the AD/*APOE* ε4+ participants might also play a role in altering Eotaxin-3 levels. In parallel, two other biomarkers, Leptin and PYY, have shown increased levels in the AD/*APOE* ε4+ subgroup at both time points, strongly suggesting that in the AD/*APOE* ε4+ subgroup, these analytes may represent a stable biomarker alternative in the *APOE* ε4− carrier individuals, while seeking for a biomarker panel sufficient for AD detection. It is interesting to note that both Leptin and PYY perform similar functions as they affect the metabolism by reducing appetite after a meal. A study in *APOE* ε4 knock-in mice has shown that Leptin receptors are specifically reduced in the hippocampal neurons, similar to the levels observed in Tg2576 mice, but not in *APOE* ε3 knock-in mice^39^. This suggests that *APOE* ε4 carriers may develop some resistance to Leptin signalling and that increased levels of Leptin could be a consequence of this altered metabolism. Similarly, NPY receptor levels (the receptor for PYY) were reportedly lower in the cortex and in the hippocampus of AD brains^40^, although it is not known whether their levels are affected by *APOE* genotype.

While finding a biomarker panel that can differentiate between HC and AD remains a primary target, another important goal is to predict the development of AD. This would allow interventions before onset of the disease. In order to determine if this can be achieved with our approach, we evaluated our biomarkers with respect to the Aβ deposition in the brains of HC participants. Our analysis therefore tested the association of biomarker levels with the neocortical SUVR score rather than specific areas. Since the neocortical regions are the most commonly affected by Aβ deposition in AD^41^, the detection of Aβ using this approach should be more consistent.

Because it is understood that this deposition starts years before the clinical manifestation of the disease and it is accepted that HC with high amyloid load are more likely to progress to AD in the future^10^, we subdivided this group into PiB− (SUVR < 1.5, low amyloid deposition) and PiB+ (SUVR > 1.5, high amyloid deposition). When the biomarker levels were analysed with this stratification we found that at 54 months, IL-12/23p40 levels were significantly higher in PiB+ HC than the PiB−, while the earlier 18 month assessment only exhibited a trend. IL-10 levels also followed this trend at both time points in PiB+ compared to PiB−, while all other biomarkers did not display any association with amyloid load.

Consistent with studies in mice, we found that IL-10 and IL-12/23p40 are jointly associated with the amyloid deposition in the brain, indicating that several factors are involved in this process^42,43^. IL-12 is a heterodimeric cytokine produced by a number of cells associated with the immune system with a broad range of activities and acts on T- and natural killer cells. It shares the p40 subunit which is also common to IL-23^44^ and has been previously associated with AD. IL-12 is expressed by activated macrophages which serves as an inducer for T-helper cell (Th1) development^45^, which have important roles in the adaptability of the immune system and its regulation. At present, the connection of the IL-12/23p40 subunit with AD is not well understood. However, polymorphisms of the gene encoding it have been associated with autoimmune diseases^46–48^ and susceptibility for multiple sclerosis^49–54^, particularly those that increase its production^55^.

Reduced levels seems to lower the inflammatory response, as seen with mice deficient in the p40 subunit which developed less severe forms of autoimmune diseases^56^. It is not known if this is consistent in the brain, but it was demonstrated in other mouse models that inhibition of IL-12/23 p40 was associated with reduced Aβ levels^57,58^. This seems to agree with a recent small scale study using CSF from human participants that showed a positive correlation of IL-12/23 p40 with CSF Aβ1-42^59^, and our own current work here. This evidence strengthens the connection between brain amyloid load and the immune system.

While there is still clearly much to learn about this link, a clue may lie with the cytokines sharing the p40 subunit. IL-12 production from whole blood after mitogen stimulation was found to be lower in cells from AD patients compared to healthy controls^60^. Another study showed IL-12 levels were initially elevated in mild AD and decreased as the disease progressed^61^. These findings indicate that while IL-12 and other cytokines may play an early role in development of AD, the capacity of cells to produce these cytokines is diminished with disease progression, indicating a reduced immune response in AD^62^. This reduction of IL-12 during disease progression appears to be generalised and not confined to the blood and periphery, since IL-12 levels were also found to be lower in the CSF of AD patients^63^.

IL-23, the other heterodimeric cytokine which shares the p40 subunit is important in the inflammatory response during infection, affecting both innate and adaptive immune system functions^64,65^. Inflammation is considered a feature of AD pathology and this cytokine is known to promote inflammatory responses, such as upregulation of the matrix metalloprotease MMP9^56^. It was even considered to be a primary cytokine for autoimmune inflammation in the brain^66^.

Like IL-12, not much is actually known about its primary role in the brain. However, many studies have reported associations with AD. IL-23 levels appeared to be increased in AD^67^, which may be a direct consequence of the increased numbers of IL-23-producing cells in AD^68,69^. As IL-12 and IL-23 displayed different modulation in AD, it brings into question the roles of the other subunits of these two interleukins, namely p35 and p19 for IL-12 and IL-23, respectively. Regardless, both IL-10 and IL-12/23p40 may be considered as viable biomarkers in a broader panel to help identify individuals at risk of developing AD, where early therapeutic intervention may delay the onset of the disease. Overall, IL-12 and IL-23 are considered pro-inflammatory cytokines mainly secreted by antigen-presenting cells, such as dendritic cells and macrophages, with extensive roles in in the immune response and the differentiation of Th1 and Th17 subset of T-helper cells^44,66,70–73^. Conversely, IL-10 is one of the more potent anti-inflammatory cytokines which inhibits the secretion of Th1 cytokines from stimulated cells^74–77^.

A problem that may arise from this kind of analysis is the extreme heterogeneity of the studies. Various biomarker panels have given different results based on the different proteins and methods used in the evaluation. Even when the same proteins were examined in different studies, their involvement in AD was inconsistent. Furthermore, the sources of these analytes is important, i.e. whether it is CSF or blood-derived specimens. Inconsistencies across different studies may be due to a combination of factors, including different assay platforms but particularly the stage of the disease, as several proteins may be affected during early or late stages in AD. Although blood was typically drawn from fasting individuals, the time of the day is another factor that should be considered as many proteins have a day-night cycle that severely affects their levels over the daily cycle. The statistical analysis used to determine the significance of the biomarker can play a role in determining which analytes are significant and which are not. Finally, the number of times blood is drawn and examined can be a very important factor as it would provide a more detailed and regular analysis, eliminating the risk of false positive/false negative results that can easily occur during a single time point analysis.

However, in our study we tried to address these issues and detected two biomarkers that are jointly associated with brain amyloid deposition in the HC that can be used in a broader biomarker panel for the detection of individuals at higher risk of developing AD. As it is increasingly necessary to identify individuals at risk for AD, such findings would allow for early treatments with the ultimate goal to stop or delay the onset of the disease.

## Electronic supplementary material

Supplementary Table
