# Supplementary material for: A blood-based biomarker panel indicates IL-10 and IL-12/23p40 are jointly associated as predictors of β-amyloid load in an AD cohort
Source: Sci Rep. 2017 Oct 25;7:14057. doi: 10.1038/s41598-017-14020-9 (PMC5656630; doi:10.1038/s41598-017-14020-9)
Supplement: Supplementary file 1 — Supplementary Table [file 41598_2017_14020_MOESM1_ESM.doc]

**A blood-based biomarker panel indicates IL-10 and IL-12/23p40 are jointly associated as predictors of β-amyloid load in a AD cohort.**

Steve Pedrini^1,2#^, Veer B. Gupta^1,2#^, Eugene Hone^1,2#^, James Doecke^3^, Sid O’Bryant^4^, Ian James^5^, Ashley I. Bush^2,6^, Christopher C. Rowe^7^, Victor L. Villemagne^7^, David Ames^8,9^, Colin L. Masters^6^, Ralph N. Martins^1,2,10,11^*, and the AIBL Research Group^12^

1. School of Medical Sciences, Edith Cowan University, Joondalup, WA 6027, Australia.
2. Co-operative Research Centre for Mental Health, http://www.mentalhealthcrc.com
3. CSIRO Digital Productivity Flagship, Brisbane, QLD, 4029, Australia
4. University of North Texas health Science Center, Fort Worth, Texas, USA
5. Institute for Immunology & Infectious Diseases, Murdoch University, Australia.
6. The Florey Institute, The University of Melbourne, Parkville, Victoria, 3052, Australia.
7. Department of Nuclear Medicine and Centre for PET, Austin Health, Heidelberg, Victoria, 3084, Australia.
8. National Ageing Research Institute, Parkville, Victoria, 3052, Australia.
9. Academic Unit for Psychiatry of Old age, St. George’s Hospital, The University of Melbourne, Victoria, 3101
10. School of Psychiatry and Clinical Neurosciences, University of Western Australia
11. Department of Biomedical Sciences, Faculty of Medicine and Health Sciences, Macquarie University, Sydney, NSW 2109, Australia
12. www.aibl.csiro.au

Supplementary Table 1

|  | HC 18 | AD 18 | HC 54 | AD 54 |
| --- | --- | --- | --- | --- |
| EGF | 27.89 ± 1.71 | 28.61 ± 6.42 | 17.45 ± 1.15 | 22.37 ± 2.10 |
| IL12/23 p40 | 117.89 ± 3.30 | 120.43 ± 8.59 | 122.74 ± 3.59 | 138.18 ± 8.85 |
| IL-15 | 1.96 ± 0.02 | 2.14 ± 0.07 | 2.26 ± 0.03 | 2.59 ± 0.09 |
| IL-17 | 1.52 ± 0.08 | 2.11 ± 0.26 | 1.82 ± 0.33 | 2.05 ± 0.18 |
| MCP-1 | 62.44 ± 0.90 | 68.78 ± 5.41 | 67.58 ± 1.08 | 82.91 ± 8.99 |
| MMP-2 | 83578 ± 758 | 86777 ± 2666 | 89272 ± 897 | 93380 ± 2595 |
| Eot-3 | 5.03 ± 0.67 | 7.94 ± 2.92 | 6.83 ± 2.61 | 6.44 ± 1.79 |
| IL-5 | 0.69 ± 0.06 | 0.47 ± 0.05 | 0.60 ± 0.04 | 0.51 ± 0.05 |
| IL-6 | 1.31 ± 0.24 | 1.41 ± 0.20 | 1.68 ± 0.35 | 1.44 ± 0.14 |
| IL-7 | 0.98 ± 0.05 | 0.90 ± 0.11 | 0.64 ± 0.02 | 0.76 ± 0.06 |
| IL-10 | 1.32 ± 0.14 | 1.05 ± 0.05 | 1.25 ± 0.11 | 1.42 ± 0.27 |
| TARC | 71.95 ± 4.73 | 76.36 ± 9.78 | 55.65 ± 2.82 | 69.28 ± 5.74 |
| TNF-α | 1.62 ± 0.03 | 1.85 ± 0.09 | 1.71 ± 0.03 | 1.99 ± 0.10 |
| Ang-2 | 8042 ± 179 | 9.38 ± 698 | 8894 ± 211 | 9652 ± 612 |
| SCF | 92.71 ± 1.17 | 92.12 ± 3.70 | 98.11 ± 1.37 | 99.28 ± 3.40 |
| EGFR | 34315 ± 297 | 33540 ± 789 | 34538 ± 307 | 32706 ± 685 |
| Leptin | 27968 ± 1855 | 30331 ± 4902 | 31053 ± 2098 | 38482 ± 5875 |
| PYY | 87.44 ± 1.95 | 104.95 ± 7.20 | 95.01 ± 2.15 | 102.38 ± 6.31 |

Supplementary Table 1

Average levels of all biomarkers in HC and AD at 18 and 54 month time points, including all samples that were excluded in the previous analysis due to high % CV. Data are presented as Mean ­± S.E..
